# Matrix-bound nanovesicles prevent ischemia-induced retinal ganglion cell axon degeneration and death and preserve visual function

**DOI:** 10.1038/s41598-019-39861-4

**Published:** 2019-03-05

**Authors:** Yolandi van der Merwe, Anne E. Faust, Ecem T. Sakalli, Caroline C. Westrick, George Hussey, Kevin C. Chan, Ian P. Conner, Valeria L. N. Fu, Stephen F. Badylak, Michael B. Steketee

**Affiliations:** 1 Department of Bioengineering, University of Pittsburgh, Pittsburgh, PA USA; 2 Department of Ophthalmology, University of Pittsburgh, Pittsburgh, PA USA; 3 McGowan Institute for Regenerative Medicine, University of Pittsburgh, Pittsburgh, PA USA; 4 Department of Molecular Biology, Bogazici University, Istanbul, Turkey; 5 Department of Surgery, University of Pittsburgh, Pittsburgh, PA USA; 6 Center for Neuroscience, University of Pittsburgh, Pittsburgh, PA USA

**Keywords:** Innate immune cells, Cellular neuroscience, Visual system

## Abstract

Injury to retinal ganglion cells (RGC), central nervous system neurons that relay visual information to the brain, often leads to RGC axon degeneration and permanently lost visual function. Herein this study shows matrix-bound nanovesicles (MBV), a distinct class of extracellular nanovesicle localized specifically to the extracellular matrix (ECM) of healthy tissues, can neuroprotect RGCs and preserve visual function after severe, intraocular pressure (IOP) induced ischemia in rat. Intravitreal MBV injections attenuated IOP-induced RGC axon degeneration and death, protected RGC axon connectivity to visual nuclei in the brain, and prevented loss in retinal function as shown by histology, anterograde axon tracing, manganese-enhanced magnetic resonance imaging, and electroretinography. In the optic nerve, MBV also prevented IOP-induced decreases in growth associated protein-43 and IOP-induced increases in glial fibrillary acidic protein. *In vitro* studies showed MBV suppressed pro-inflammatory signaling by activated microglia and astrocytes, stimulated RGC neurite growth, and neuroprotected RGCs from neurotoxic media conditioned by pro-inflammatory astrocytes. Thus, MBV can positively modulate distinct signaling pathways (e.g., inflammation, cell death, and axon growth) in diverse cell types. Since MBV are naturally derived, bioactive factors present in numerous FDA approved devices, MBV may be readily useful, not only experimentally, but also clinically as immunomodulatory, neuroprotective factors for treating trauma or disease in the retina as well as other CNS tissues.

## Introduction

Central nervous system (CNS) neurons often fail to regenerate after injury, leading to lost neurologic function. In the visual system, damage to retinal ganglion cells (RGCs), CNS neurons in the retina that send visual information via the optic nerve (ON) to the brain, often results in vision loss due to progressive RGC axon degeneration^1^. RGC degeneration is regulated in part by a pro-inflammatory innate immune response in resident glia^2^. After retina or optic nerve injury, microglia are hypothesized to polarize toward a pro-inflammatory, M1-like, phenotype and secrete, among other factors, tumor necrosis factor-α (TNF-α), interleukin-1α (IL-1α), and complement protein C1q^3^. These factors, in turn, are thought to act directly^4^ and indirectly on RGCs by inducing an A1-like, neurotoxic phenotype in astrocytes that signals RGC axon degeneration and death^5^.

Porcine-derived extracellular matrix (ECM) bioscaffolds are immunomodulatory biomaterials that have been used successfully in various tissue engineering, regenerative medicine, and general surgery applications^6^, with over 60 FDA approved ECM-based products available clinically and over 8 million patients treated to date. ECM bioscaffolds are most commonly xenogeneic in origin and are prepared by decellularizing pro-regenerative source tissues like dermis, urinary bladder, and small intestinal submucosa (SIS), among others^7^. Xenogeneic bioscaffolds have been shown to support positive tissue remodeling over scarring in all major tissue types, including muscle, epithelial, connective, and even nervous system tissues. When properly prepared, ECM bioscaffolds do not elicit an adverse innate or adaptive immune response. In fact, positive tissue remodeling is strongly linked to the ability of ECM bioscaffolds to promote an anti-inflammatory, M2-like, innate immune response^8^.

Recently, matrix-bound nanovesicles (MBV) were identified as critical bioactive factors within ECM bioscaffolds^9^. MBV are a distinct class of extracellular vesicle localized to collagen fibrils within the ECM of all experimental and commercial ECM bioscaffolds analyzed to date^9^. In addition to their ECM specific localization, MBV are distinct from other extracellular vesicles, like exosomes^10^, based on their lipid profiles, membrane-associated proteins, and unique nucleic acid and protein cargo^9,11,12^. MBV can deliver cargo to diverse cell types, and purified MBV can recapitulate many of their parent ECM’s effects, including polarizing cells underlying the innate immune response toward an anti-inflammatory phenotype^13^ and differentially regulating primary CNS neuron survival and growth^14^.

This study reports on the effects of MBV on primary microglia, astrocytes, and RGCs *in vitro* and, on RGC viability, RGC axon integrity and connectivity to visual nuclei in the brain, and visual function after severe intraocular pressure (IOP) induced ischemia. Given the clinical relevance of porcine-derived ECM materials^15^ and the well-studied characteristics of decellularized porcine urinary bladder matrix (UBM)^16^, MBV derived from UBM extracellular matrix (UBM-ECM) were used in this study. *In vitro*, MBV suppressed the release of pro-inflammatory cytokines from primary microglia and astrocytes and neuroprotected primary RGCs cultured in neurotoxic media conditioned by pro-inflammatory astrocytes. *In vivo*, intravitreally injected MBV prevented RGC axon degeneration, RGC death, and preserved retinal ganglion cell-dependent visual function after acute IOP elevation in rat. This study’s results support the hypothesis that MBV can positively modulate the default healing response in the retina and in the optic nerve in part by modulating the innate immune response to reduce neurotoxic, pro-inflammatory glial signaling. Moreover, in contrast to extracellular vesicles derived from *in vitro* sources, this study shows extracellular vesicles, derived from natural, readily available xenogeneic tissues, can be used to positively modulate the default healing response in CNS tissues like the retina and optic nerve.

## Results

### MBV differentially regulate RGC neurite growth and viability *in vitro*

**Figure 1:**
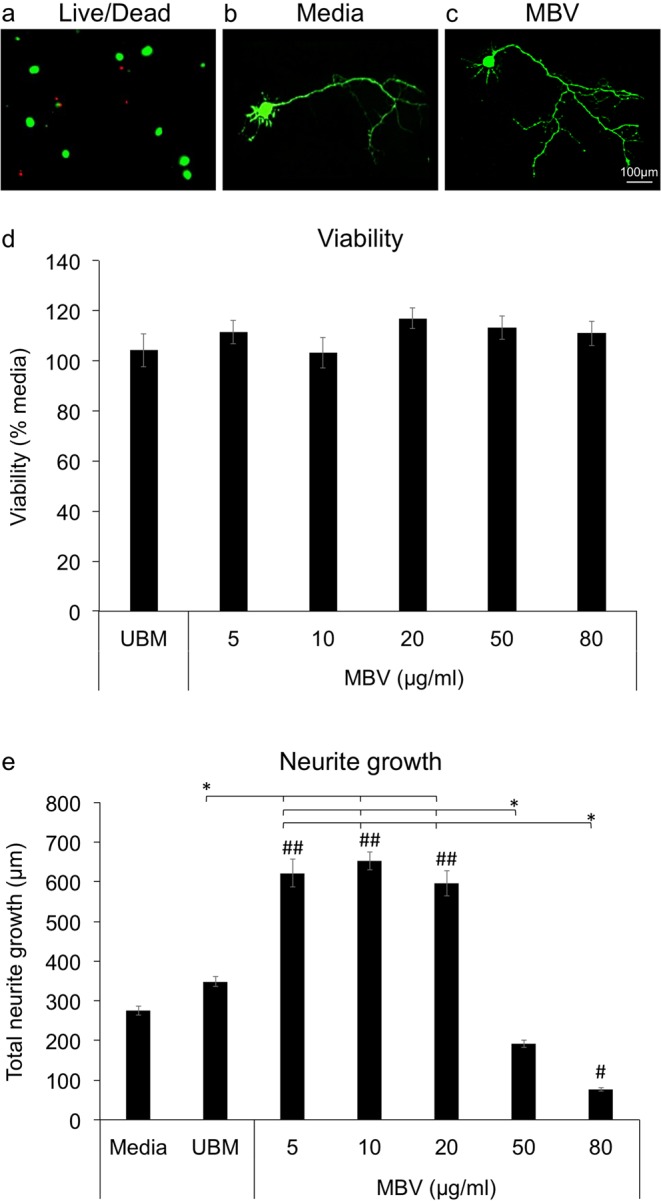
MBV increase RGC neurite growth. (**a**) RGC live/dead analysis. Green (calcein) and red (propidium iodide) indicate live and dead cells, respectively. (**b**,**c**) Representative images show individual RGCs in (**b**) Media and (**c**) Media with MBV. (**d**) Normalized to media alone, RGC viability was unchanged by either UBM-ECM (250 µg/ml) or MBV (5–80 µg/ml). (**e**) Total RGC neurite growth increased over 5–20 µg/ml before decreasing dose-dependently to below control levels at 80 µg/ml. (**d**,**e**) Error bars indicate the SEM, n > 300 neurons analyzed from 3 independent experiments. Significance was determined by one-way ANOVA between groups, ^#^*p* < 0.01, ^##^*p* < 0.001 compared to media and *p < 0.01 between groups.

To determine if MBV influence RGC survival and growth, we analyzed RGC viability (Fig. 1a) and total neurite growth (Fig. 1b,c) in control media, media with MBV, ranging in concentration from 5 to 80 µg/ml, and media with UBM-ECM at 250 µg/ml, based on the growth of hippocampal neurons^14^, which have been shown to predict RGC neurite growth *in vitro*^17^. MBV did not change viability over the concentration range tested (Fig. 1d). In contrast, MBV changed total neurite growth in a concentration-dependent manner (Fig. 1e). Compared to total neurite growth in media, which averaged 275.2 ± 11.6 µm, or in media with UBM-ECM, which averaged 348.3 ± 12.1 µm, MBV increased total neurite growth at 5, 10, and 20 µg/ml to 621.5 ± 35.2 µm, 652.4 ± 23.2 µm, and 596.0 ± 32.3 µm, respectively. However, at 50 µg/ml, total neurite growth was similar to control lengths, averaging 191.6 ± 9.9 µm, and shorter than control lengths at 80 µg/ml, averaging 76.4 ± 4.3 µm. Thus, total neurite growth in primary RGCs exhibits a bi-phasic dose response to MBV and, similar to studies with other CNS neurons *in vitro*^14^, MBV can regulate RGC neurite growth independent of viability.

### MBV suppress pro-inflammatory glial signaling

**Figure 2:**
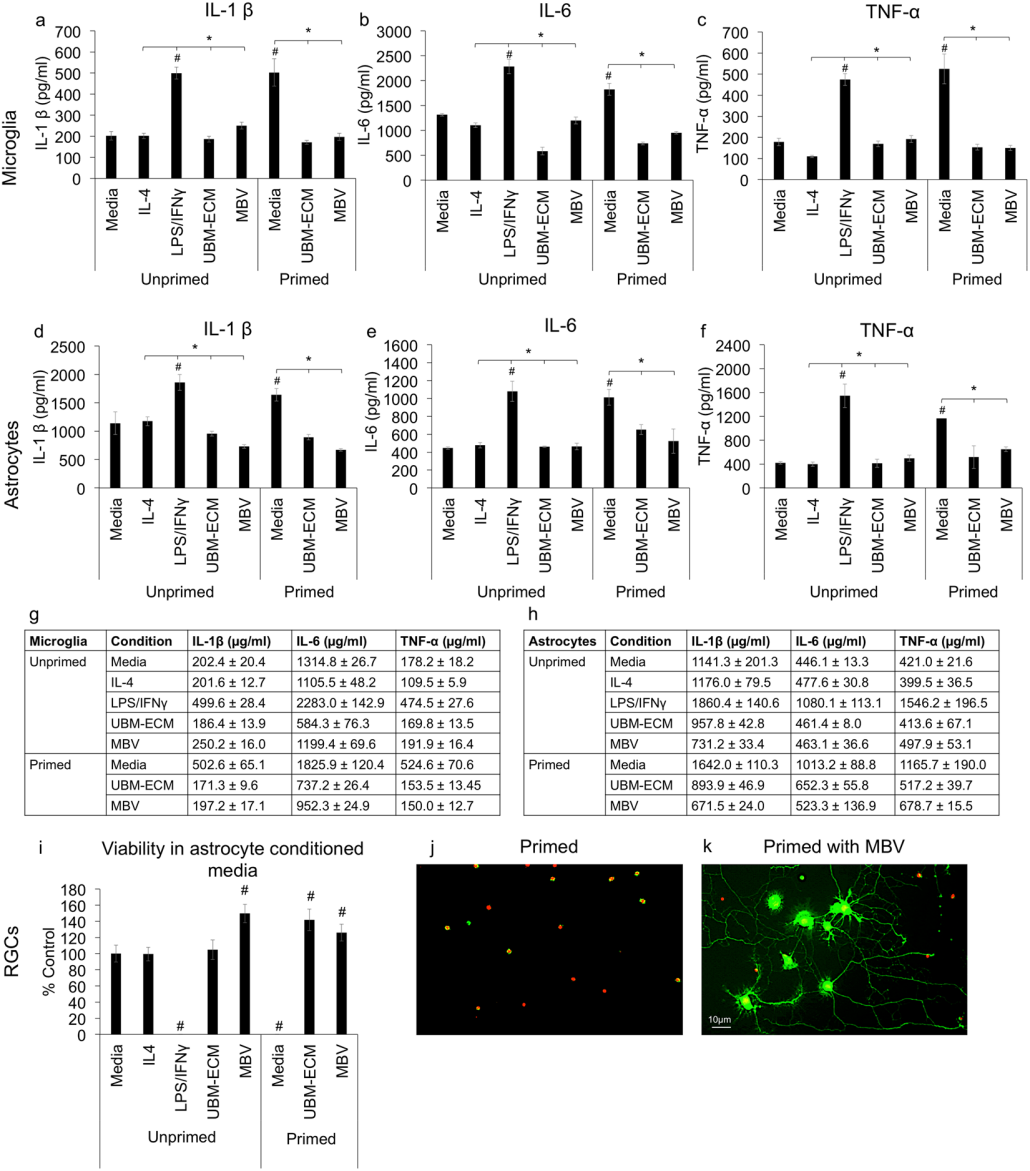
MBV decrease pro-inflammatory cytokine secretion from glia and neuroprotect RGCs. (**a**–**c**) In unprimed microglia, LPS/IFNγ increased IL-1β, IL-6, and TNF-α secretion. In LPS/IFNγ primed microglia, IL-1β, IL-6, and TNF-α secretion increased in media, but both UBM-ECM (250 µg/ml) and MBV (5 µg/ml) neutralized these increases. (**d**,**f**) Similar to microglia, LPS/IFNγ increased IL-1β, IL-6, and TNF-α secretion from unprimed astrocytes. Media conditioned by primed microglia also increased IL-1β, IL-6, and TNF-α but UBM-ECM and MBV neutralized these increases. (**g**,**h**) Tables show the mean ± SEM for the (**g**) Microglia and (**h**) Astrocyte data in graphs (**a**–**f**) Data represent triplicates from three independent experiments. (**i**–**k**) RGCs were cultured in media conditioned by unprimed or primed astrocytes. (**i**) After 3 DIV, RGCs treated with either LPS/IFNγ in unprimed media or primed media alone showed 100% RGC death. RGC cultures treated with unprimed media with MBV or primed media with UBM-ECM or MBV had increased numbers of viable cells compared to control. (**h**,**i**) Representative live (green) and dead (red) images of RGCs in primed astrocyte media (**j**) without or (**k**) with MBV. Data represent n > 300 neurons analyzed from 3 independent experiments normalized to viability in unconditioned media. Error bars indicate the SEM. One-way ANOVA determined significance between groups, *p < 0.05, and compared to unconditioned media, ^#^p < 0.01.

Since UBM-ECM can promote an alternatively activated, anti-inflammatory phenotype in microglia and astrocytes^18^, we analyzed whether MBV derived from UBM-ECM can also regulate pro-inflammatory cytokine signaling in microglia and astrocytes (Fig. 2). Initially, MBV effects on pro-inflammatory cytokine secretion by microglia were determined (Fig. 2a–c). In unprimed microglia, LPS/IFNγ increased IL-1β, IL-6, and TNF-α secretion compared to media alone, whereas IL-4, UBM-ECM (250 µg/ml), and MBV (5 µg/ml) did not. The concentration of IL-1β, IL-6, and TNF-α increased in microglia cultures primed with LPS/IFNγ for 24 hrs and then cultured in fresh control media, indicating that pro-inflammatory cytokine secretion was sustained in primed microglia. However, both UBM-ECM and MBV decreased the release of all three pro-inflammatory cytokines compared to control, unprimed levels.

Since activated microglia are hypothesized to activate and induce pro-inflammatory signaling in astrocytes^19^, we analyzed pro-inflammatory cytokine secretion from primary rat astrocytes treated with conditioned media (CM) from either unprimed or primed microglia cultures. For primed microglia CM, microglia were pre-primed with LPS/IFNγ for 6 hours, washed, and then cultured in fresh media for 24 hours, with the resultant CM added to astrocyte cultures (Fig. 2d–f). Compared to unprimed microglia CM and IL-4, both primed microglia CM and LPS/IFNγ (positive control) increased IL-1β, IL-6, and TNF-α secretion. In contrast, primed microglia CM treated with either UBM-ECM or MBV failed to increase the secretion of all three pro-inflammatory cytokines. Thus, CM from pro-inflammatory microglia can stimulate astrocytes to increase pro-inflammatory cytokine secretion, and this effect is neutralized by both UBM-ECM and MBV.

### MBV neuroprotect RGCs *in vitro*

Next, the ability of MBV to neuroprotect RGCs from neurotoxic astrocyte signaling was analyzed. Normalized to the number of viable RGCs in astrocyte conditioned nb-SATO media (unprimed CM), which averaged 1125 ± 114 RGCs/mm^2^, RGC viability was 99.5% (1119 ± 95 RGCs/mm^2^) in unprimed CM with Il-4, 0% (0 RGCs/mm^2^) in unprimed CM with LPS/IFNγ, and 104.8% (1176 ± 144 RGCs/mm^2^) in unprimed CM with UBM-ECM. In contrast, MBV increased RGC viability in unprimed CM to 149.7% (1685 ± 132 RGCs/mm^2^). In nb-SATO conditioned by primed astrocytes (primed CM), RGC viability decreased to 0% (0 RGCs/mm^2^). However, both UBM-ECM and MBV prevented this decrease. In primed CM with UBM-ECM, RGC viability was 141.9% (1597 ± 153 RGCs/mm^2^) and 126.4% (1422 ± 123 RGCs/mm^2^) in primed CM with MBV. Together, these results support the hypothesis that signaling from activated microglia can regulate RGC viability indirectly by inducing pro-inflammatory signaling in astrocytes and show that MBV can neutralize neurotoxic signaling from pro-inflammatory astrocytes.

### MBV neuroprotect RGCs *in vivo*

**Figure 3:**
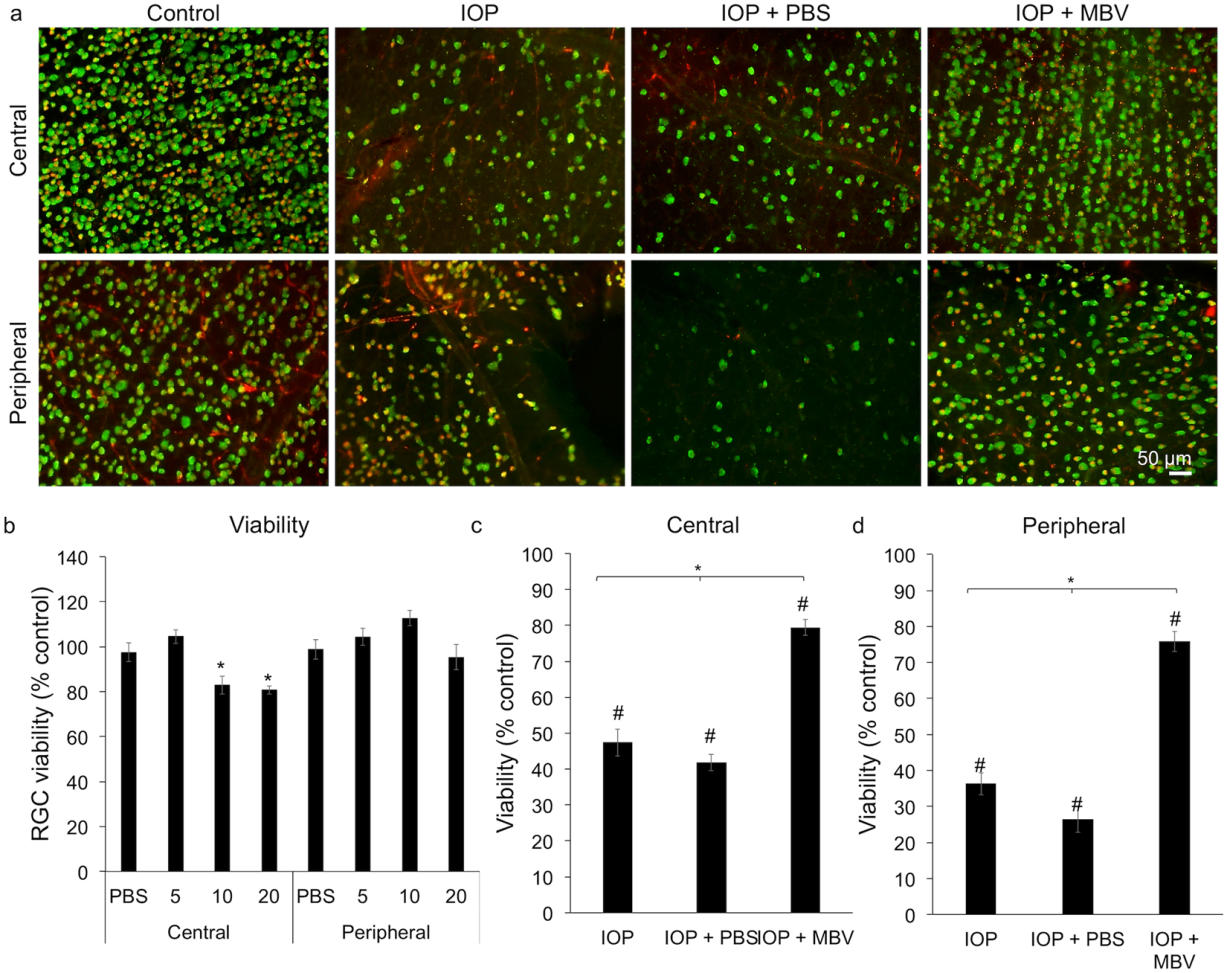
MBV decrease IOP-induced RGC death. (**a**) Representative images show RGC cell bodies co-labeled with RBPMS (Green) and Brn3A (Red) in the uninjured central and peripheral retina (Control), in the IOP-injured retina (IOP), and in IOP-injured retinas treated with either PBS (IOP + PBS) or MBV (IOP + MBV). (**b**) To determine *in vivo* toxicity, MBV were injected intravitreally at 5, 10, or 20 µg/ml, on days 0, 2, and 7 into healthy, uninjured rat eyes and analyzed at 14 days. Compared to uninjected controls, RGC viability was unchanged by either PBS or MBV at 5-µg/ml. However, viability was  reduced in the central but not in the peripheral retina by 10- and 20-µg/ml MBV injections at 14 days. (**c**,**d**) In IOP-injured eyes, MBV decreased IOP-induced RGC loss in both the (**c**) Central and in the (**d**) Peripheral retina compared to IOP-injured and IOP-injured with PBS injections. Data represent n = 5 animals per group and 12 images per retina, totaling 60 images per group. Error bars indicate the SEM. One-way ANOVA with Post-hoc Tukey’s test determined significance between groups, *p < 0.05, and compared to uninjured control, ^#^*p* < 0.05.

To determine if MBV can also neuroprotect RGCs *in vivo*, MBV were injected intravitreally into both the uninjured left eye and the right eye injured by elevating IOP from 15 to 130 mmHg for 60 min. (Fig. 3). In uninjured retinas, anti-RBPMS and -Brn3a co-localization showed typical RGC labeling patterns in the central and in the peripheral retina (Fig. 3a, control). In both untreated and PBS treated IOP-injured eyes, RBPMS and Brn3a were markedly reduced qualitatively compared to uninjured controls, both in the central and in the peripheral retina, consistent with widespread RGC loss. In contrast, MBV injections preserved RBPMS and Brn3a positive RGCs both in the central and in the peripheral retinas of IOP-injured eyes. In uninjured eyes, MBV dose-response results over 5-20 µg/ml (Fig. 3b) indicated RGC viability was similar to control both in the central (104.4 ± 4.1%) and in the peripheral (104.2 ± 5%) retina at an injection concentration of 5 µg/ml. At 10 and 20 µg/ml, RGC viability decreased to 82.8 ± 1.9% and 80.7 ± 4.4% respectively in the central retina (Fig. 3b). Therefore, MBV were  injected at 5 µg/ml into IOP-injured eyes with untreated IOP-injured eyes and PBS injected IOP-injured eyes serving as controls. Based on the time course for RGC death in similar IOP-dependent injury models^20^ and an estimated 1-3 day half-life for nanometer-sized vesicles in the vitreous^21,22^, MBV or PBS vehicle were injected on day 0, immediately after IOP injury, and on days 2 and 7, with animal sacrifice on day 14.

Quantitative analyses complimented qualitative observations. Compared to uninjured controls, IOP injury reduced RBPMS and Brn3a co-localization in the central retina to 47.4 ± 2.3% of controls. Similarly, RBPMS and Brn3a were reduced to 41.8 ± 2.3% in the retinas from PBS treated, IOP-injured eyes. However, RBPMS and Brn3a only decreased to 79.5 ± 3.8% of controls in MBV treated, IOP-injured eyes (Fig. 3c). We observed similar results in the peripheral retina. Compared to uninjured controls, RBPMS and Brn3a decreased to 36.3 ± 3.6% in IOP-injured retinas and to 26.4 ± 2.8% in IOP-injured retinas treated with PBS, whereas RBPMS and Brn3a immunoreactivity in the retinas from MBV treated eyes averaged 75.9 ± 4.2% (Fig. 3d). Thus, MBV neuroprotected RGCs in both the central and in the peripheral retina *in vivo*, preserving approximately 80% of the RGCs compared to uninjured controls.

### MBV prevent IOP-induced axon degeneration

**Figure 4:**
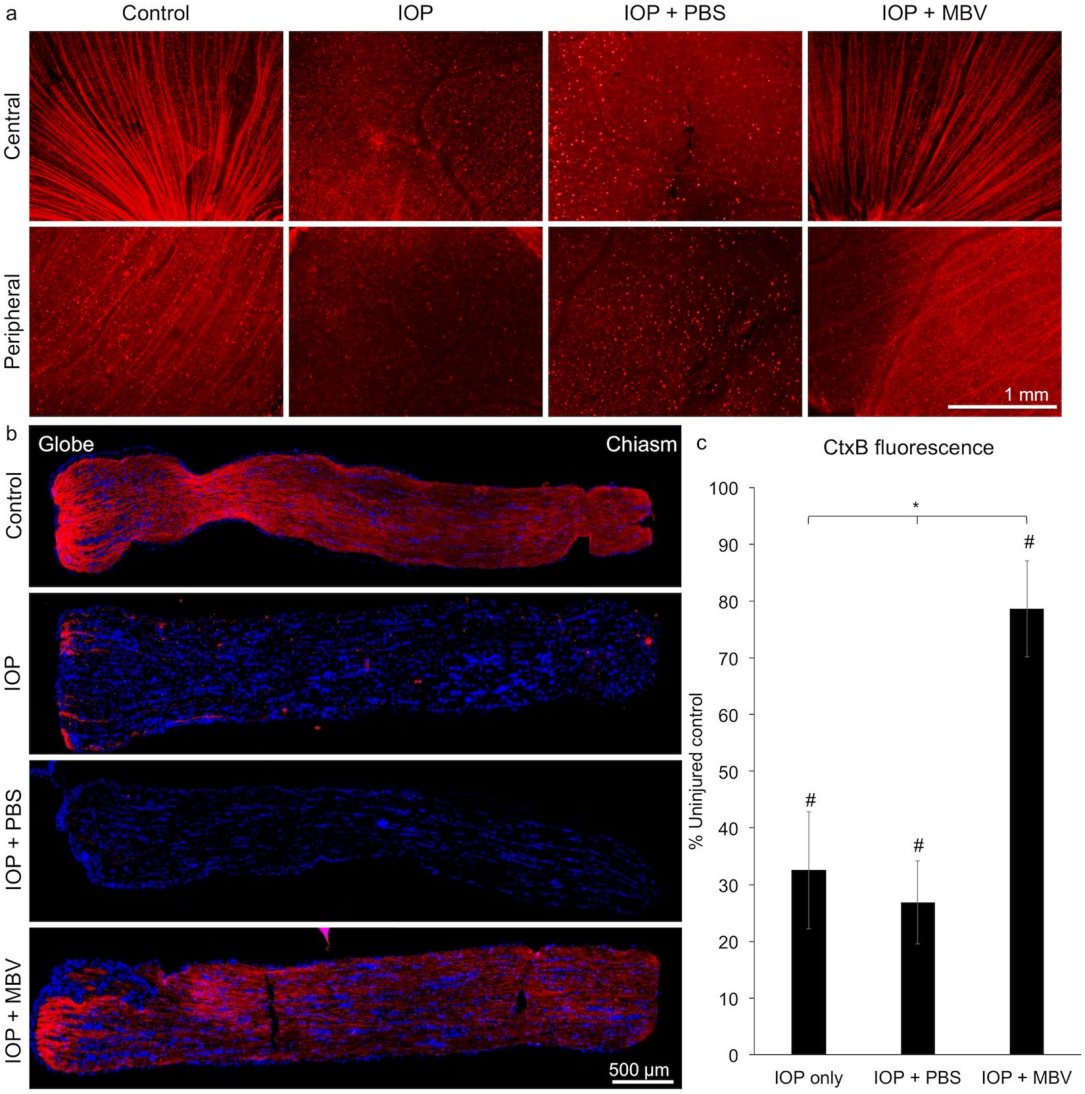
MBV decrease IOP-induced axon degeneration. (**a**) Representative images showing RGC axons labeled with cholera toxin subunit B (CTB, red) in the uninjured central and peripheral retina (Control), in IOP-injured retina (IOP), and IOP-injured retinas treated with either PBS (IOP + PBS) or MBV (IOP + MBV). (**b**) Representative images showing RGC axons labeled with CTB in the optic nerve (ON) of uninjured control eyes (Control), untreated IOP-injured eyes (IOP), and IOP-injured eyes treated with either PBS (IOP + PBS) or MBV (IOP + MBV). (**c**) Quantitatively, CTB immunofluorescence decreased in both untreated and PBS treated IOP-injured ONs. However, in MBV treated IOP-injured ONs, CTB was more similar to uninjured control animals. Data represent n = 5 animals per group and 15 images per ON, totaling 75 images per group. Error bars indicate the SEM. One-way ANOVA with Post-hoc Tukey’s test determined significance between groups, *p < 0.05, and compared to uninjured control, ^#^*p* < 0.05.

To visualize RGC axons, the retrograde tracer, cholera toxin subunit B (CTB), was injected into the posterior chamber of uninjured and IOP-injured eyes treated with either PBS or MBV (Fig. 4). In uninjured retinas, CTB revealed a typical, radially organized pattern, both in the central and in the peripheral retina, consistent with intact RGC axon fascicles extending toward the optic nerve head. In contrast, CTB labeling in the retinas from both untreated and PBS treated, IOP-injured eyes revealed highly disorganized CTB positive puncta, consistent with widespread RGC axon degeneration. In retinas from MBV-treated, IOP-injured retinas, CTB labeling was qualitatively similar to uninjured controls. In the uninjured ON, CTB revealed linear fascicles traversing the length of the ON (Fig. 4b). In untreated and PBS treated IOP-injured ONs, CTB positive fascicles were qualitatively undetectable (Fig. 4b, IOP and IOP + PBS). However, in MBV treated, IOP-injured ONs, CTB labeling was similar to uninjured control ONs, with linear fascicles extending the length of the ON (Fig. 4b, IOP + MBV), consistent with intact RGC axons. Quantitation confirmed qualitative observations (Fig. 4c). In untreated and PBS treated IOP-injured ONs, CTB immunofluorescence decreased to 32.5 ± 6.3% and 26.8 ± 4.4% of controls respectively. In IOP-injured ONs, MBV increased CTB to 78.6 ± 6.1% of control ONs, over 2-fold higher than in untreated and PBS treated IOP-injured ONs. Thus, MBV mitigate IOP-induced RGC axon degeneration in the ON.

### MBV preserved GAP-43 expression

**Figure 5:**
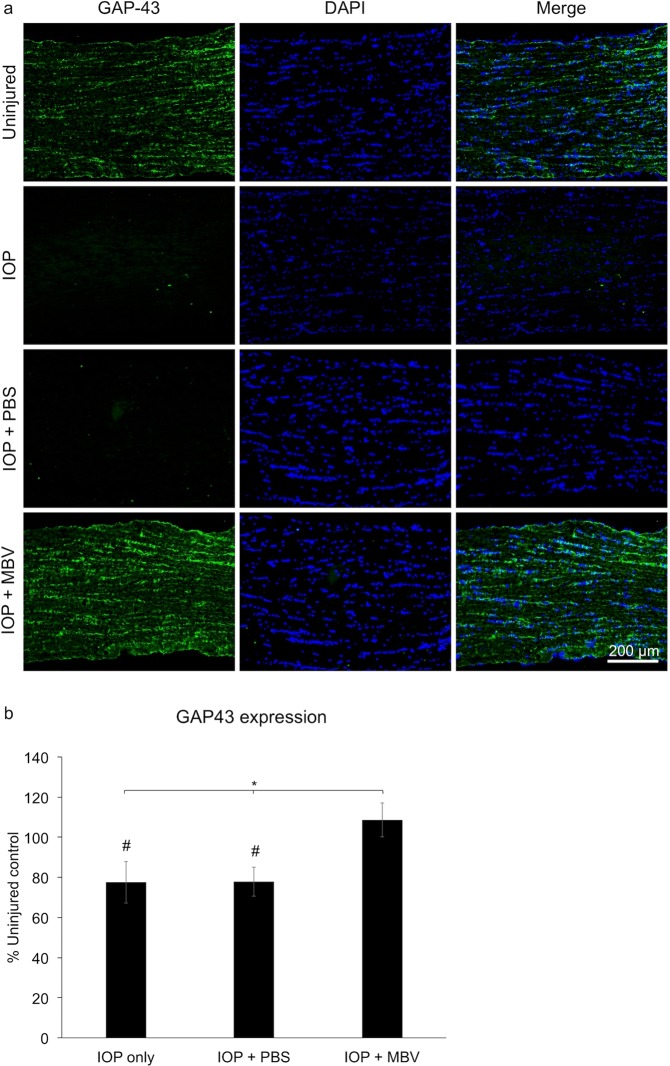
MBV prevent IOP-induced decreases in GAP-43 expression in the optic nerve. (**a**) Representative images showing GAP-43 expression and DAPI positive nuclei in the optic nerves, 2 mm posterior to the globe, of uninjured control eyes (control), untreated IOP-injured eyes (IOP), and IOP-injured eyes treated with either PBS (IOP + PBS) or MBV (IOP + MBV). (**b**) Quantitatively, MBV injections prevented IOP-induced decreases in GAP-43 immunoreactivity. Data represent n = 5 animals per group and 15 images per optic nerve, totaling 75 images analyzed per group. Error bars indicate the SEM. One-way ANOVA with Post-hoc Tukey’s test determined significance between groups, *p < 0.05, and compared to uninjured control, ^#^*p* < 0.05.

To further visualize RGC axons in the optic nerve, growth-associated protein-43 (GAP-43) immunoreactivity was analyzed (Fig. 5a). GAP-43 is a neuron-specific marker up regulated in developing^23^ and in regenerating axons^24^. However, low-level GAP-43 expression in mature RGCs provides an RGC axon specific marker in the ON. Compared to uninjured ONs, GAP-43 immunoreactivity decreased in untreated and PBS treated, IOP-injured ONs, averaging 77.5 ± 7.2% and 77.8 ± 8.4% of controls, respectively (Fig. 5b). In MBV treated, IOP-injured ONs, GAP-43 immunoreactivity was similar to uninjured ONs, averaging 108.6 ± 6.1%. These results complement the CTB tracing data by showing MBV treatment can prevent IOP-induced loss in GAP-43 expression, which is consistent with functionally intact RGC axons.

### MBV prevented IOP-induced increases in GFAP expression

**Figure 6:**
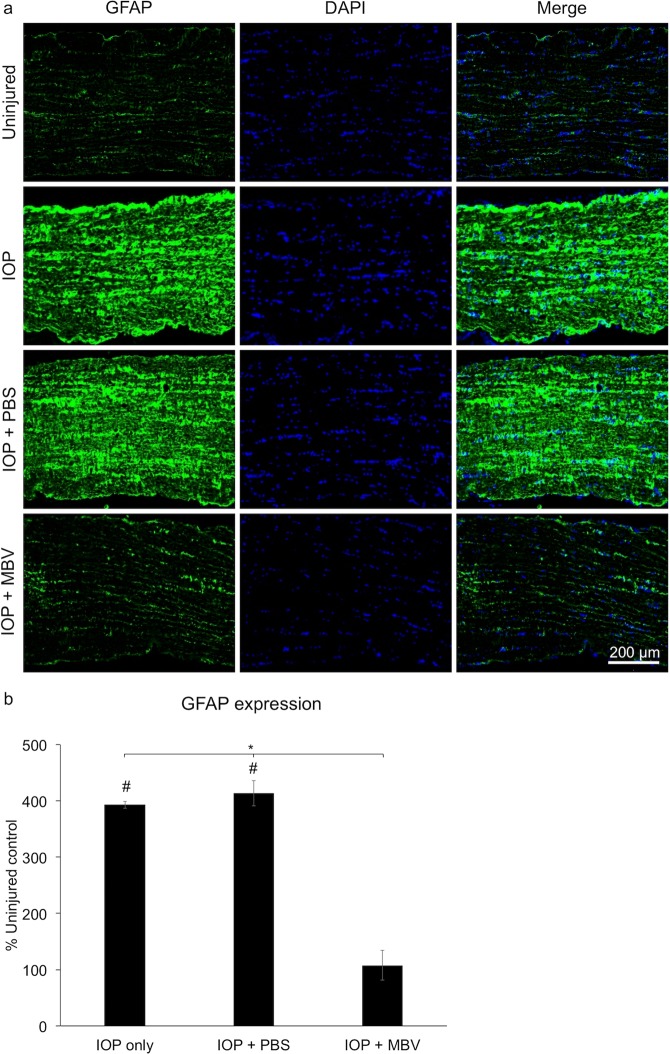
MBV prevent IOP-induced increases in GFAP expression in the optic nerve. (**a**) Representative images showing GFAP expression and DAPI positive nuclei in the optic nerve, 2 mm posterior to the globe, of uninjured control eyes (control), untreated IOP-injured eyes (IOP), and IOP-injured eyes treated with either PBS (IOP + PBS) or MBV (IOP + MBV). (**b**) Quantitatively, MBV (IOP + MBV) but not PBS (IOP + PBS) injections prevented IOP-induced increases in GFAP immunoreactivity in the optic nerve of IOP-injured eyes. Data represent n = 5 animals per group and 15 images per optic nerve, totaling 75 images per group. Error bars indicate the SEM, One-way ANOVA with Post-hoc Tukey’s test determined significance between groups, *p < 0.05, and compared to uninjured control, ^#^*p* < 0.05.

Since astrocyte activation is a marker for RGC axon health and pathology^25^, we analyzed glial fibrillary acidic protein (GFAP) immunoreactivity in the ONs of IOP-injured eyes with or without MBV treatment (Fig. 6A). Compared to uninjured ONs, GFAP increased qualitatively in untreated and PBS treated IOP-injured ONs, consistent with activated astrocytes reacting to degenerating RGC axons^26^. Quantitatively, normalized GFAP expression increased to 393 ± 22.7% in untreated and to 413.7 ± 26.3% in PBS treated IOP-injured ONs, whereas GFAP expression in MBV treated IOP-injured ONs was similar to uninjured controls, averaging 107.7 ± 6.4% (Fig. 6B). These results are consistent with MBV-dependent mitigation of IOP-induced axon degeneration.

### MBV preserved RGC axon connectivity to visual nuclei

**Figure 7:**
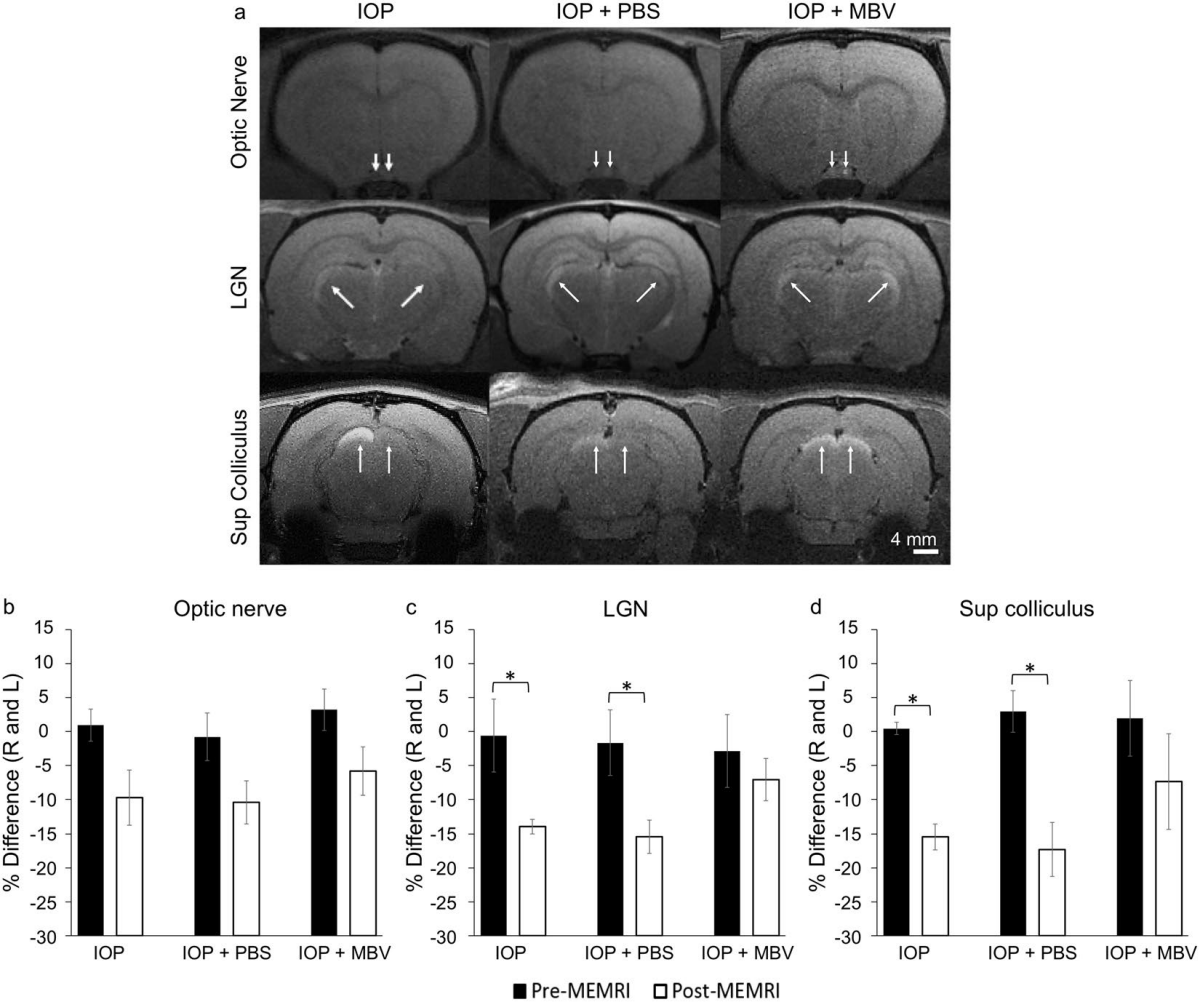
MBV preserve RGC axon connectivity to visual nuclei in the brain. (**a**) Representative manganese-enhanced MRI (MEMRI) images of the optic nerve, lateral geniculate nucleus, and superior colliculus (SC) in IOP-injured eyes treated with either PBS (IOP + PBS) or MBV (IOP + MBV). Manganese signal intensities (arrows) were normalized to a saline phantom to control for system instability. Percent differences in manganese-enhanced axonal transport were calculated between the right and left visual projections in the (**b**) Optic nerve, (**c**) Lateral geniculate nucleus (LGN), and in the (**d**) Superior colliculus (SC). Data represent n = 5 animals per group. Error bars indicate the SEM. One-way ANOVA with Post-hoc Tukey’s test determined significance between pre- and post-MEMRI. *p < 0.05

Manganese (Mn) enhanced magnetic resonance imaging (MEMRI) was used to investigate the functional integrity and connectivity of RGC axons to visual nuclei in the brain. Mn transport by RGC axons was visualized from the left and right retinas to the lateral geniculate nuclei (LGN) and superior colliculi (SC) (Fig. 7) in live rats. In the ON, differences between the right and left signal intensities in the pre- and post-MEMRIs were undetectable for all three groups (Fig. 7A). In contrast, the differences in the LGN signal intensities in the untreated and PBS treated, IOP-injured groups were significantly lower (IOP Pre: 0.6 ± 5.3% and Post: 13.9 ± 1.1%; IOP + PBS Pre: 1.7 ± 4.8 and post: 15.4 ± 2.5%), whereas there was no significant difference in the signal intensities in the MBV treated, IOP-injured group (IOP + MBV Pre: 2.9 ± 5.3 and Post: 7.1 ± 3.1%; Fig. 7B). Similarly, the signal intensities in the SC were lower in both the untreated and PBS treated, IOP-injured groups (IOP Pre: −0.4 ± 0.9% and Post: 15.5 ± 1.9%; IOP + PBS Pre: −2.9 ± 3.1 and IOP + PBS Post: 17.3 ± 4.0%) but not in the SC of the MBV treated, IOP-injured group (IOP + MBV Pre: −2.0 ± 5.6 and IOP + MBV Post: 7.3 ± 7.1%; Fig. 7C). These data indicate RGC axons from MBV treated, IOP-injured retinas actively transported Mn along the entire length of the visual pathway, consistent with intact RGC axons terminated within visual nucleiin the brain.

### MBV preserved retinal function

**Figure 8:**
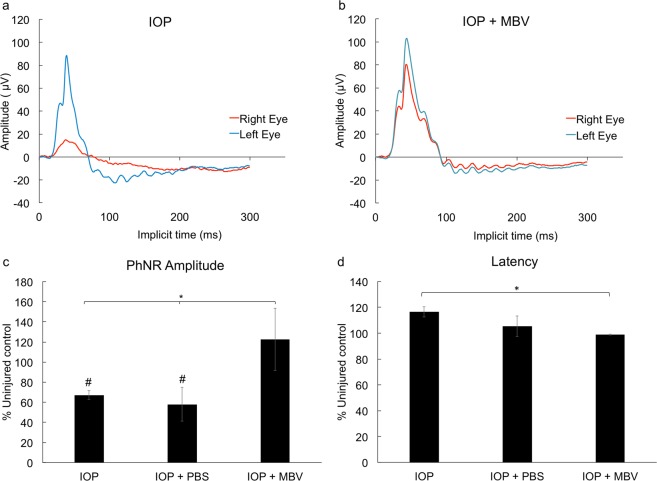
MBV preserve RGC-dependent retinal function. Representative electroretinography traces from a left (uninjured) and a right (IOP-injured) retina 14 days after (**a**) IOP elevation (IOP) and (**b**) IOP elevation with MBV treatment (IOP + MBV). (**c**) Quantitatively, IOP elevation, with or without PBS control injections, decreased the photopic negative response (PhNR) compared to uninjured control retinas. In contrast, the PhNR amplitude was similar to uninjured controls in IOP + MBV treated retinas. (**d**) In IOP-injured retinas (IOP), the PhNR latency increased compared to IOP + MBV treated retinas but not uninjured controls. Data represent n = 5 animals per group and 3 ERG recordings per animal. Error bars indicate the SEM. One-way ANOVA with Post-hoc Tukey’s test determined significance between groups, *p < 0.05, and compared to uninjured control, ^#^*p* < 0.05.

Electroretinography (ERG) was then used to determine if MBV prevent IOP-induced loss in retinal function. ERG recorded RGC-dependent retinal activity from the left, uninjured and the right, IOP-injured eye. The photopic negative response (PhNR) amplitude and latency were recorded to analyze the RGC contribution to the ERG full field response (Fig. 8). Representative ERG responses for IOP-injured only and MBV treated, IOP-injured eyes are shown (Fig. 8A,B). Compared to uninjured control eyes, the PhNR amplitude decreased by 32.8 ± 4.4% in IOP-injured eyes and by 42.0 ± 16.8% in PBS treated, IOP-injured eyes, whereas there was no difference in PhNR amplitude between uninjured and MBV treated eyes (Fig. 8C). PhNR latency was unchanged in all three groups compared to uninjured controls. However, PhNR latency in IOP-injured eyes increased by 16.6 ± 4.0% compared to MBV treated IOP-injured eyes (Fig. 8D).

## Discussion

This study shows MBV derived from porcine UBM-ECM can suppress pro-inflammatory signaling in both microglia and astrocytes and neuroprotect RGCs both *in vitro* and *in vivo* after acute IOP elevation in rat by documenting several new findings. First, injecting MBV intravitreally prevented ischemia-induced RGC loss and preserved RGC-dependent retinal function. As in previous studies^27^, elevating IOP to 130 mmHg for 60 minutes lead to over 50% RGC loss by 14 days. In contrast, viability was approximately 80% of control in MBV treated retinas, over 2-fold higher than in control retinas. Notably, whether MBV injections permanently spared RGCs in IOP-injured retinas or whether MBV simply slowed the rapid RGC die-off phase^20^ remains to be determined over more prolonged endpoints. Moreover, MBV dose and regimen also require optimization to maximize efficacy and minimize MBV induced toxicity. In this study, MBV was administered at 5-μg/ml since higher MBV concentrations, 10 and 20 μg/ml, reduced RGC viability in the central retina specifically. Though the mechanism is unknown, MBV were injected into the vitreous over the optic nerve head. As in humans, RGCs in the rodent central retina, are more susceptible to specific types of injury, like ischemia, than RGCs in the periphery^28^, likely due in part to differences in RGC subtype, function, and retinal anatomy. Thus, ongoing studies are analyzing MBV uptake sites and pharmacokinetics to optimize MBV injection concentration, location, and frequency.

Differences in RGC viability were reflected by differences in RGC-dependent retinal function as analyzed by ERG. As in previous studies^29^, IOP-induced ischemia led to a significant decline in the PhNR, a component of the full-field flash ERG specific for RGC-dependent retinal activity^30^. MBV injections prevented the IOP-induced loss in PhNR similar to previous studies using stem cell-derived exosomes^31^. Whether these ERG data reflect preserved functional vision remains under investigation in pigmented rats since optokinetic analysis of visual behavior in Sprague-Dawley rats is unreliable due to their inherently low visual acuity^32^.

Second, MBV prevented IOP-induced RGC axon degeneration from the retina to visual nuclei in the brain. In IOP-injured animals, linear RGC axon fascicles were virtually undetected qualitatively in the ON by either CTB anterograde tracing or GAP-43 immunoreactivity and GFAP expression, a marker for reactive astrocytes, was elevated throughout the optic nerve. Non-reactive astrocytes provide critical structural, physiologic, and metabolic support to RGC axons^33^. However, after injury, reactive astrocytes and increased GFAP expression are consistent features of IOP-induced RGC axon degeneration^26^. Thus, the observed increases in GFAP in IOP-injured ONs are consistent with widespread IOP-induced RGC axon degeneration.

Conversely, in MBV treated, IOP-injured ONs, RGC axon fascicles in the retina and the ON were similar to uninjured controls with radially organized CTB positive fasciculi in the retina becoming linearly arranged tracks traversing the length of the ON. GAP-43 and GFAP were also similar to uninjured controls, consistent with the MBV-dependent prevention of IOP-induced RGC axon degeneration and consequently low levels astrocyte activation^34^. However, whether MBV deliver neuroprotective cargos directly to glia or to RGCs *in vivo* or whether MBV positively modulate the phenotypes of other cellular populations, like neutrophils and macrophages, to neuroprotect RGCs indirectly remains under investigation.

MEMRI complimented and extended the histology data by showing that RGC axons maintained connectivity from the retina to visual nuclei in the brain in MBV treated IOP-injured animals. In MBV, but not untreated or PBS treated IOP-injured animals, Mn transport was visible from the posterior globe to both the LGN and the SC in the visual cortex. Since Mn transport requires active transport by intact RGC axons^35^, these data show that functional RGC axons remain terminated within their appropriate visual nuclei 14-days post-injury. Though additional time points and optokinetic testing are required, the combined histology, MRI, and ERG data suggest MBV injections preserved visual function in IOP-injured animals.

This study’s *in vivo* results compliment and extend previous studies documenting RGC neuroprotection in both trauma and disease models. Similar to acute ischemia, optic nerve crush (ONC) induces rapid RGC death over the first two weeks post injury followed by slower progressive RGC loss, consistent with the activation of similar pro-apoptotic, injury-signaling pathways in both models. After ONC, RGCs have been neuroprotected to varying degrees by pro-inflammatory stumulii^36,37^, or modulating the intrinsic growth ability of RGC axons alone^17,38^ or in combination with extrinsic factors^39–41^. Similarily, progressive RGC death and axon degeneration can be slowed or even prevented in induced and genetic glaucoma models by acetylcholine receptor agonists^42,43^ or phosphodiesterase inhibitors^44^. However, although ONC and induced or genetic glaucoma models, like the DBA/2J mouse, mimic many features of ocular traumas or diseases, these models are generally not relevant clinically since they do not mimic typical ocular trauma or disease etiologies^45^. Moreover, many of the experimental interventions are not readily translatable or have not been as neuroprotective in humans as in rodents^46^. In contrast to the above studies, the acute ischemia model used in this study mimics acute arterial occlusion seen clinically in humans, which is also characterized by local ischemia-reperfusion, secondary trauma, progressive neurodegeneration, and permanent vision loss.

Moreover, although the exact targets have not yet been identified, MBV deliver combinatorial factors that positively modulated the injury response across multiple immune, glial, and neuronal cell types. Our findings are consistent with other studies showing that injections of mesenchymal stem cell derived extracellular vesicles (MSC-EV) can also attenuate the immune response, preserve retinal function, and neuroprotect RGCs in several rodent disease and injury models, including experimental autoimmune uveitis^47^, type 1 diabetes rodent^48^, glaucoma^49,50^, laser induced inflammation^51^ and optic nerve crush^31^ models. However, although the findings in these studies are promising and consistent with our observations, none of these studies showed the degree of RGC neuroprotection documented in this study. Moreover, acquiring MSC-EV is inherently problematic due the necessary surgical procedures, rigorous purification steps and precise culturing conditions required to obtain consistent bioactive MSC-EV. In contrast to MSC-EV, MBV are naturally derived factors that are inexpensive, readily available, and highly translatable since they are derived from the same ECM bioscaffolds currently used in over 60 FDA approved devices.

The ability of MBV to prevent RGC axon degeneration supports the hypothesis that MBV block injury responsive signaling generated in the cell body and/or proximal axon necessary to initiate anterograde, pro-degenerative signaling in the distal axon. Though a thorough review of injury-induced, pro-degenerative signaling in neurons is beyond the scope of this study, MBV provide new tools for determining how proximally injured neurons signal axon degeneration distally as well as testing canonical neuronal death signaling pathways in mammalian neurons such as the dual leucine zipper kinase (DLK) dependent activation of c-Jun via c-Jun N-terminal kinase (JNK)^52^ and, in turn, the JNK-dependent regulation of SARM1 mediated axonal degeneration^53^, among other somatic and axonal injury signaling pathways in neurons^54^.

Third, MBV neuroprotected RGCs from neurotoxic astrocyte signaling. Previous studies showed MBV could shift macrophages toward an anti-inflammatory, M2-like, phenotype, recapitulating the effects of UBM-ECM on macrophages^55^. This study extends those findings by showing MBV can also shift primary rat microglia and astrocytes toward anti-inflammatory phenotypes; MBV suppressed pro-inflammatory cytokine secretion from both microglia and astrocytes. Moreover, when MBV were added directly to RGCs cultured in neurotoxic astrocyte conditioned media, the number of viable RGCs increased from 0% to 126% of control, indicating MBV can suppress neurodegenerative signaling either by acting directly on RGCs or indirectly by neutralizing the effects of neurotoxic factors secreted by reactive astrocytes.

Notably, in unprimed astrocyte-conditioned media, MBV were more efficacious than UBM-ECM. This observation highlights an important mechanistic difference between MBV and the parent ECM that is important to consider when interpreting physiological responses to ECM and ECM derived biomaterials. Unlike purified MBV, both the structural and soluble components of UBM-ECM contribute to the overall cellular response through distinct mechanisms^56^. MBV were also more efficacious in unprimed, astrocyte-conditioned media than in unconditioned control media. A wide variety of metabolic and biochemical cofactors can differentially regulate extracellular vesicle activities, including cell entry, trafficking, and cargo bioactivities. Thus, the molecular profile and the biochemical and cellular context in which naturally derived bioactive factors, like extracellular vesicles, are studied are important to take into consideration when interpreting results.

One example is the bi-phasic regulation of RGC neurite growth by MBV *in vitro*. At lower concentrations, MBV increased neurite growth; however, at higher concentrations, MBV decreased neurite growth. Studies have documented bi-phasic neurite growth responses in cultured RGCs and other primary neurons^57^. One plausible explanation is that different MBV cargos regulate distinct mechanisms or signaling pathways underlying neurite growth. Similar to other extracellular vesicles^58^, MBV carry distinct protein and nucleic acid cargos, including highly conserved miRNAs, e.g., miRNAs-30b, -125b, and -133b, known to regulate neuronal differentiation and neurite growth^59^. A second example is the differential regulation of RGC viability *in* vitro and *in vivo*, whereby MBV were non-toxic *in vitro* but exhibited RGC toxicity in the central but not the peripheral retina *in vivo*. Thus, ongoing studies are focused on more detailed biochemical and molecular analyses of MBV uptake, cargo delivery, and the direct and indirect effects of MBV cargo bioactivities in relevant cellular populations *in vitro* and *in vivo*.

An important caveat is that MBV cargo profiles, and thus bioactivities, likely depend on the properties of the source tissue. Based on protein and RNA analyses^9^ and consistent phenotypes in recipient cell types^13,14^, the MBV used in this study appear to represent a tissue-specific population with similar bioactivities when derived from similarly sourced, healthy porcine urinary bladders. However, whether MBV derived from the ECMs of different, pro-regenerative tissues, like dermis, small intestine mucosa, or urinary bladder, modulate the innate immune response similarly remains to be determined. As with other extracellular vesicles, MBV cargos likely change with the source tissue’s species, age, and health, among other factors. Thus, identifying the bioactive factors in MBV and analyzing how and when bioactive MBV cargos change in response to various environmental stimuli and how such changes, in turn, regulate cellular phenotypes is necessary to advance MBV technology. Furthermore, since small nanometer-sized particles or vesicles, like MBV, have relatively short half-lives in the vitreous^22^, identifying the bioactive factors mediating the positive effects of MBV are likely necessary to develop effective, sustained delivery devices for treating chronic diseases.

MBV can be derived from FDA approved, commercially available ECM bioscaffolds successfully used in millions of patients, and therefore hold a low risk of adverse side effects clinically. Their stability, nano-scale size, and engineerability to carry additional bioactive factors^14^ make MBV good candidates for diverse, minimally invasive applications either alone or incorporated into existing devices.

## Materials and Methods

### Animal care and use

Sprague-Dawley rats were from Charles River Laboratories (Wilmington, MA). Animal care followed the Guide for the Care and Use of Laboratory Animals published by the National Institutes of Health and all experimental protocols were approved by the University of Pittsburgh Institutional Animal Care and Use Committee.

### UBM-ECM and Matrix-bound nanovesicles (MBV)

Porcine urinary bladders were decellularized, analyzed, and digested with pepsin to form UBM-ECM pre-gels (10 mg/ml) as described^60^. MBV were prepared by digesting lyophilized UBM-ECM with Liberase TL (5401020001, Sigma-Aldrich Corp., St. Louis, MO) as described^9^. MBV concentration was determined using a Pierce BCA Protein assay kit (23225, ThermoFisher Scientific) and MBV purity and morphology were analyzed qualitatively and quantitatively by randomly measuring MBV diameters in electron micrographs acquired at 80 kV with a JEOL 1210 transmission electron microscope (JEOL, Peabody, MA) as described^9^.

### RGC purification, viability, and neurite growth

RGCs were isolated from postnatal day three Sprague-Dawley pups as described^61^ and seeded immediately in neurobasal-SATO (nb-SATO) media at 3,000 RGCs/well in 96-well plates (Falcon 087723B, Corning Inc, Corning, NY) coated with poly-D-lysine (70 kDa, 10 μg/ml, Sigma-Aldrich Corp., St. Louis, MO) and laminin (2 μg/ml, L-6274, Sigma-Aldrich Corp., St. Louis, MO). RGCs were cultured at 37 °C, 10% CO_2_ for 3 days with MBV (0-80 µg/ml) or UBM-ECM (250 μg/ml). Using a Live/Dead Imaging Kit (R37601, Life Technologies, Carlsbad, CA), the number of viable RGCs per area was quantified^14^. Five random, non-overlapping fields were imaged at 20×, totaling 45 fields of view. For total neurite growth, RGCs were labeled with anti-βIII tubulin (1:300, TUJ-1, MAB5564, Millipore, Burlington, MA). The first ten RGCs encountered, not contacting another RGC, were measured, totaling 90 RGCs per group, as described^62^.

### Microglia

Primary rat microglia, from Lonza (R-G-535, Lonza, Switzerland), were plated at 50,000 cells/well in 96-well plates in microglia media (Lonza, Switzerland) at 37 °C, 5% CO_2_ per instructions. For unprimed cultures, microglia were treated with: microglia media, lipopolysaccharide (LPS, 100 ng/ml, 297-473-0, Sigma-Aldrich, St. Louis, MO) and interferon gamma (IFNγ, 20 ng/ml, 50-919-6, Fisher Scientific, Hampton, NH), interleukin-4 (20 ng/ml, 130-097-761, Miltenyi Biotech, Germany), UBM-ECM (250 µg/ml), or MBV (5 µg/ml). For primed cultures, microglia were treated with LPS/IFNγ for 6 hrs, washed (1×), and incubated in microglia media with or without UBM-ECM (250 µg/ml) or MBV (5 µg/ml). After 24 hrs, microglia-conditioned medias (CM) were collected and analyzed by ELISA or added to astrocyte cultures (see below).

### Astrocytes

Primary rat astrocytes (R1800, ScienCell Research Laboratories, Carlsbad, CA) were cultured in astrocyte media (ScienCell Research Laboratories) at 5,000 cells/well for 24 hrs at 37 °C, 5% CO_2_ per instructions. After 24 hrs, the astrocytes were washed and cultured in either a microglia CM or control media. After 24 hrs, the astrocytes were washed (1×) and then incubated in fresh astrocyte media for 24 hrs. The astrocyte CMs were added to RGC cultures or analyzed by ELISA for IL-1β (DY501, R TNF-α (438204, Biolegend, San Diego, CA), IL-6 (DY506 R&D Systems, Minneapolis, MN), and TNF-α (438204, Biolegend, San Diego, CA). Data represent duplicates from three experimental repeats.

### RGC viability in astrocyte CM

Astrocytes were cultured in transwell inserts (CLS3397, Sigma-Aldrich) at 5,000 cells/well in astrocyte media. After 24 hrs, the media was replaced with microglia CM for 24 hrs, washed with nb-SATO (1×), and then incubated in nb-SATO for 24 hrs. The astrocyte-conditioned nb-SATO was collected and added to RGC cultures for 72 hrs. RGC viability was analyzed as described above. All RGC and glial cultures were monitored visually and counted manually to assure similar numbers of healthy cells per well after plating and before and after priming or adding experimental factors.

### Intraocular pressure elevation

IOP was elevated in the right eye of forty-five Sprague-Dawley rats as described^63^. Briefly, ketamine: xylazine anaethesia (75:10 mg/kg) was administered intraperitoneally and eyes received one drop each of proparacaine and tropicamide to induce analgesia and pupil dilation, respectively. A 30 g needle, connected to a saline reservoir (0.9% sodium chloride; Baxter International Inc., Deerfield, IL), was inserted into the anterior chamber parallel to the iris and secured in place. The reservoir was elevated to increase the IOP to 130 mmHg for 60 min. IOP was measured using a pressure transducer (BIOPAC Systems, Goleta, CA, USA) and a handheld tonometer (Icare TONOLAB, Finland). After 60 min, the reservoir was lowered, the needle removed, and a drop of gentamicin applied (Akorn, Lake Forest, IL).

### Intravitreal injections

Animals were anesthetized as above and each eye received a drop of proparacaine and tropicamide. Using a Hamilton micro-syringe with magnification, MBV (5, 10, or 20-µg/ml) or PBS was injected (1-µl) intravitreally without contacting the lens or retina. After injection, IOP was equalized for 30-sec before retraction. Animal numbers were calculated using G*power software (Germany). An effect size of 0.6 indicated five animals per group to achieve 80% for α = 0.05. Fifty-seven animals were divided into nineteen per group as follows: n = 10 for histology, n = 5 for ERG, n = 4 for MEMRI. MBV were injected (1 µl, 5-µg/ml, n = 19 animals) immediately after IOP elevation, on day 0, and on days 2 and 7. CTB-Alexa Fluor 594 was injected (2 µl, 1% in PBS, C3477, Life Technologies, Carlsbad, CA) on day 11.

### Immunohistochemistry (IHC)

Retinas and ONs were fixed, sectioned, and processed for IHC as described^28^. Retinas were co-labeled with rabbit anti-RBPMS (1:250, 1830-RBPMS, Phosphosolutions, Aurora CO) and mouse anti-Brn3A (1:250, SC-8429, Santa Cruz, Santa Cruz CA) amplified with Alexa Fluor 488 donkey anti-rabbit (1:500, AB150073, Abcam, United Kingdom) or Alexa Fluor 555 goat anti-mouse (1:500, AB150114, Abcam, United Kingdom). CTB was amplified with anti-CTB (1:250, mouse anti-CTB, AB62429, Abcam, United Kingdom) followed by Alexa Fluor 555 goat anti-mouse. ON sections were labeled with anti-GAP43 (1:500, rabbit anti-GAP-43, AB16053, Abcam, United Kingdom), anti-GFAP (1:500, rabbit anti-GFAP, AB7260, Abcam, United Kingdom) and amplified as above. Sections were mounted in Vectashield (H-1200, Vector Laboratories, Burlingame, CA) and imaged with standard epi-fluorescence. For retinas, 12 peripheral and 12 central images were acquired and RBPMS and Brn3a, dual-positive nuclei were quantified per region. For ONs, the area, mean fluorescence, and integrated density were measured for fifteen regions of interest (ROIs), evenly distributed along the ON and for four ROIs on the background. The signal intensity was calculated as follows: CTCF = integrated density−(area × mean background fluorescence).

### Manganese-enhanced MRI (MEMRI)

Each animal (n = 4 per group) received a 1.5 µl bilateral injection of 100 mM MnCl_2_. T1-weighted MEMRI was done immediately before and then 8 hrs post injection with a fast spin echo imaging sequence as described^35^. Briefly, slices were oriented orthogonal to the pre-chiasmatic ON. Other imaging parameters include: TR/TE = 600/8 ms, echo train length = 8, number of slices = 8, and slice thickness = 1 mm. A saline phantom was placed next to the rodent head for normalization. Signal intensity values, from IOP-injured and uninjured animals, before (pre-MEMRI) and 8 hrs after (post-MEMRI) MnCl_2_ injections were calculated as percentage differences between the right and left signal intensities and compared between pre- and post-MEMRI to determine the percent decrease in intensity in the ON, LGN, and SC.

### Electroretinography (ERG)

Retinal function was recorded by ERG as described^61^. Briefly, animals were anesthetized with ketamine/xylazine (IP, 75:10 mg/kg) and one drop of proparacaine and tropicamide was applied to each eye. Eyes were then lubricated with Goniovisc (2.5%, 9050, Sigma Aldrich Corp, St. Louis, MO) and two gold loop electrodes placed on the cornea. The reference electrode was inserted into the cheek and the ground lead electrode into the quadriceps. Bilateral ERG recordings were made simultaneously using a color-light dome. A fixed intensity light was illuminated for 1 ms, and the ERG response recorded as a sweep over multiple steps of increasing illumination. Fifty ERG responses were recorded per trial with a total of three trials per light intensity step. Data were analyzed by measuring the PhNR and the implicit time of the different waves recorded.

### Statistical analysis

Experimentally blinded individuals analyzed data. Unless noted, one-way analysis of variance (ANOVA) and post-hoc Tukey’s test was used to determine significance between groups with *p* < 0.05.

### Ethical approval and informed consent

Sprague-Dawley rats were procured from Charles River Laboratories (Wilmington, MA). Animal care and experimental protocols complied with the University of Pittsburgh Institutional Animal Care and Use Committee and followed guidelines from the Guide for the Care and Use of Laboratory Animals published by the National Institutes of Health.

## Data Availability

The data that support the findings of this study are available from the corresponding author upon reasonable request and will be placed in a data repository as necessary.
