# Author Correction: Matrix-bound nanovesicles prevent ischemia-induced retinal ganglion cell axon degeneration and death and preserve visual function

**DOI:** 10.1038/s41598-019-50829-2

**Published:** 2019-10-29

**Authors:** Yolandi van der Merwe, Anne E. Faust, Ecem T. Sakalli, Caroline C. Westrick, George Hussey, Kevin C. Chan, Ian P. Conner, Valeria L. N. Fu, Stephen F. Badylak, Michael B. Steketee

**Affiliations:** 1 Department of Bioengineering, University of Pittsburgh, Pittsburgh, PA USA; 2 Department of Ophthalmology, University of Pittsburgh, Pittsburgh, PA USA; 3 McGowan Institute for Regenerative Medicine, University of Pittsburgh, Pittsburgh, PA USA; 4 Department of Molecular Biology, Bogazici University, Istanbul, Turkey; 5 Department of Surgery, University of Pittsburgh, Pittsburgh, PA USA; 6 Center for Neuroscience, University of Pittsburgh, Pittsburgh, PA USA

Correction to: *Scientific Reports* 10.1038/s41598-019-39861-4, published online 05 March 2019

Kevin C. Chan was omitted from the author list in the original version of this Article. This has been corrected in the PDF and HTML versions of the Article.

The Author Contributions statement now reads:

Experimental design: Y.V.d.M., A.E.F., G.H., K.C.C., S.F.B., M.B.S. Conduct experiments: Y.V.d.M., A.E.F., E.T.S., C.C.W., G.H., I.P.C., V.L.N.F. Data analysis: Y.V.d.M., A.E.F., V.L.N.F. Manuscript writing: Y.V.d.M., A.E.F., S. F. B., M.B.S.. All authors read and approved the final manuscript.

